# Preventing Pain and Stress-Related Ill-Health in Employees: A 6-Months Follow-Up of a Psychosocial Program in a Cluster Randomized Controlled Trial

**DOI:** 10.1007/s10926-022-10074-3

**Published:** 2022-10-29

**Authors:** Hedvig Zetterberg, Christiana Owiredua, Pernilla Åsenlöf, Rebecca Lennartsson, Gunilla Brodda Jansen, Katja Boersma, Steven J. Linton, Silje E. Reme, William Shaw, Michael Nicholas, Ida Flink

**Affiliations:** 1 Department of Women’s and Children’s Health, Uppsala University, Uppsala, Sweden; 2 The Center for Health and Medical Psychology, School of Law, Psychology and Social Work, Örebro University, Örebro, Sweden; 3 Division of Rehabilitation Medicine, Department of Clinical Sciences, Karolinska Institutet, Stockholm, Sweden; 4 Department of Psychology, Faculty of Social Sciences, University of Oslo, Oslo, Norway; 5 Division of Occupational and Environmental Medicine, Department of Medicine, University of Connecticut Health Center, Farmington, CT USA; 6 Pain Management Research Institute, The Kolling Institute, Sydney Medical School-Northern, University of Sydney at Royal North Shore Hospital, Sydney, Australia

**Keywords:** Chronic pain, Stress symptoms, Prevention, Randomized controlled trial, Communication, Problem solving

## Abstract

*Purpose* Pain and stress-related ill-health are major causes of long-term disability and sick leave. This study evaluated the effects of a brief psychosocial program, which previously has been tested for an at-risk population of employees. *Methods* The Effective Communication within the Organization (ECO) program, where supervisors and employees were trained in communication and problem solving, was compared to an active control consisting of psychoeducative lectures (PE) about pain and stress in a cluster randomized controlled trial. First-line supervisors were randomized to ECO or PE, and a total of 191 mainly female employees with self-reported pain and/or stress-related ill-health were included. The hybrid format programs consisted of 2–3 group sessions. Sick leave data was collected from social insurance registers, before and 6-months after the program. Secondary outcomes (work ability, work limitations, pain-disability risk, exhaustion symptoms, perceived stress, perceived health, quality of life, perceived communication and support from supervisors) were assessed at baseline, post intervention, and at 6-months follow-up. *Results* No effects were observed on primary or secondary outcome variables. Pain symptoms were common (89%), however a lower proportion (30%) were identified as at risk for long-term pain disability, which might explain the lack of evident effects. The Covid-19 pandemic affected participation rates and delivery of intervention. *Conclusion* In this study, preventive effects of the ECO program were not supported. Altogether, the findings point at the importance of selecting participants for prevention based on screening of psychosocial risk. Further research on workplace communication and support, and impact on employee health is warranted.

## Introduction

Pain and stress-related ill-health are among the largest contributors to long-term sick leave [1]. There is a need for effective interventions to prevent sick leave due to pain and stress-related ill-health. Problems with pain and stress often debut at a young age, and women are more often affected than men [2]. Interventions involving the workplace have been found to decrease sick leave more effectively than those without [3–5], hence the workplace is an important arena for interventions for stress and pain problems [3–6].

About 19–37% of adults suffer from chronic pain [2, 7], and pain conditions are associated with disability and lower work productivity [7, 8]. Problems with stress-related ill-health might be even more common; in one study, 59% of patients seeking primary health care reported some degree of stress-related problems [9]. Psychiatric comorbidity is high in chronic pain populations [2, 10] and pain and stress-related ill-health often co-occur.

This study builds on an earlier project, [11] in which a program focusing on workplace communication and problem-solving showed favorable results in terms of decreased sick leave, fewer health-care visits and better perceived health for employees with musculoskeletal low back pain, at risk for long-term pain-disability. In the current study, due to the high comorbidity between pain and stress, a similar intervention was evaluated for an extended population, embracing *both* pain and stress-related ill-health, to reduce the risk of long-term consequences.

Psychosocial factors play a key role in maintaining pain disability [12, 13]. Co-existing symptoms such as pain and stress may be explained by shared psychosocial components [14], influencing work disability, return to work [15, 16], and the risk for long-term sick leave [17]. Still, too little is known about how psychosocial factors can be managed at the workplace [6, 16, 18]. The workplace play an important role in the prevention of work disability, and specifically, supervisor interaction and support have been highlighted as potential targets for workplace interventions [6].

Prevention of pain and stress-related ill-health, disability and sick leave has clear health and economic benefits for both individual workers and their employers. The earlier mentioned study [11], focusing on workplace communication and problem-solving, provides a promising example and formed the basis for the current project. Here, we further developed the procedures and the intervention from the earlier study, with more emphasis on the supervisors, and compared this package to structured psychoeducation. A cluster randomized design was applied to avoid for supervisor to have employees in both the experimental and the control condition. The focus of this paper is on health outcomes for employees, and supervisor outcomes will be reported elsewhere [19].

The aim of this study was to compare the effects of two brief interventions in hybrid (live or web) format for employees with self-reported symptoms of pain and stress-related ill-health on sick leave and secondary health outcomes among employees, on the individual level. The psychosocial program, Effective Communication within the Organization (ECO), in which supervisors and their employees were trained in communication skills and problem solving, was compared to psychoeducational (PE) lectures. The primary outcome was amount of sick leave during 6-months follow-up (register data), the secondary health outcomes included self-reported work ability, work limitations, pain-disability risk, exhaustion symptoms, perceived stress, perceived health and quality of life, and perceived communication and support from supervisor (validation, invalidation, social support).

## Methods

### Study Design and Setting

The Prevent Sick leave (PS) project is a two-armed cluster randomized controlled trial, comparing a psychosocial prevention program for pain and stress-related ill-health, to an active control. Clusters consisted of workplace-units of supervisor (-s) and their employees, which were randomized together. Supervisors and employees were recruited via occupational health care services. Participants were assessed at baseline, immediately after the intervention, and at a 6-month follow up. The project was registered at ClinicalTrials.gov (NCT03993444) and was approved by the Regional Ethical Review Board in Uppsala, Sweden (Number 2018/479). All participants provided written informed consent prior to study participation. Recruitment was initiated in December 2018 (information to workplaces, etc.), and enrolment of participants started in March 2019. Recruitment ended September 2020. There was four intervention periods, one each semester 2019–2020. The data collection periods were April 2019 to May 2021 for questionnaires, and November 2018 to May 2021 for social insurance register data. Due to the outbreak of the Covid-19 pandemic, the project was paused March 2020 to August 2020, and a revised ethical approval to deliver the remaining intervention groups online was obtained. Follow-up time for register data was shortened from original study plan, due to the delays in the project from to the Covid-19 pandemic.

### Sample and Recruitment Procedures

The study was advertised via an occupational health care service that covers public sector workplaces such as healthcare services, schools and administrative departments. Information was sent out via email to all first-line supervisors at associated workplaces. Recruitment of employees took place at information meetings at the workplace, after inclusion of a supervisor. For employees, inclusion criteria were: (1) employed at a workplace associated to the occupational health care service, (2) self-reported pain and/or stress-related ill-health, and (3) their immediate supervisor participated in the study. Inclusion criteria for supervisors were: (1) a first-line management position at a workplace associated with the occupational health care unit, (2) personal and continuous contact with employees, and (3) time and willingness to participate in the scheduled program. Exclusion criterion for supervisors was to work less than 75% of the time, and for employees to be currently on 100% sick leave, to report an underlying non-musculoskeletal or stress-related medical condition (e.g. cancer-related pain, hyperthyroidism) affecting work ability or to suffer from severe psychiatric illness (e.g. psychosis, personality disorder).

Supervisors who expressed interest were screened to check they met inclusion criteria and received information about the study via telephone from a research coordinator. If they fulfilled the inclusion criteria and agreed to participate, informed consent was obtained and information meetings with the employees at the workplace were scheduled. The employees received information about the study and got the opportunity to ask questions of the researcher coordinator and supervisor. Volunteering employees who reported difficulties with pain and/or stress provided written informed consent when the research coordinator and the supervisor left the room, or sent them in via post in prepaid envelopes. Those who applied and fulfilled the inclusion criteria were registered in the study. Consent for social insurance data was provided separately. Supervisors were not informed about the identity, or any data from employee assessments.

### Randomization

Recruitment and randomization were organized in clusters of workplace-units, with employees and their immediate supervisor. In addition, supervisors who were linked to the same workplace were randomized together. Workplace-units were randomized in blocks of 6, in a 1:1 ratio to either the experimental or to the control (comparator) condition. Randomization was conducted by an independent researcher associated with the team. A pre-generated allocation sequence was concealed in envelopes which were opened upon enrolment of supervisors at a given workplace. To minimize participant expectations during recruitment, the same information about the study was provided to both groups and information about the interventions was sparse and manualized.

### Sample Size

A power calculation with primary outcome sick leave among employees was done to estimate sample size. Expected between group difference was a prevalence of 40% sick leave in the control group compared to 21% in the intervention group, and these numbers were based on self-reported sick leave in the previous PAIN-study [11]. With a power of 80% and alpha of 0.05 (two-tailed) a total of 182 employees was calculated as sufficient.

### Interventions

#### Effective Communication within the Organization (ECO)

The experimental group received Effective Communication within the Organization (ECO). ECO is a brief psychosocial intervention, based on a program delivered in a previous study [11]. The intervention has been revised and further developed for preventive purpose, based semi-structured interviews with personnel from human resources, supervisors and employees [20]. In the ECO supervisors and their employees are trained in communication skills and problem solving. The intervention was pilot tested in a small group of supervisors (N = 3), which resulted in minor adjustments of the protocol. An overview of the structure and content of ECO is described in Table 1. Supervisors were invited in groups of 8–10 participants, and employees in groups of 20–25 participants. Sessions followed a structured manual and were led by licensed psychologists and assisting master students in clinical psychology trained in the manual. Meetings were held at the occupational service facilities. All sessions were also available recorded, accessible through a secure web-portal together with written material and templates for home assignments. The program was modified to online video meetings, delivered live via a digital communication platform (Zoom), during autumn 2020 due to the Covid-19 situation.

**Table 1:** Overview of content in Effective Communication within the Organization (ECO)

Supervisors		Employees	
Session theme	Description	Session theme	Description
I. Communication (150 min)	Psycho-education about pain- and stress-related ill health Identification of difficulties in communication with employees, related to pain and/or stress-problems Validation and skills training in communication	I. Communication (120 min)	Psycho-education about pain- and stress-related ill-health Identification of difficulties in communication with supervisors Validation and self-validation
2 weeks to work on home assignments	Validation of others		Self-validation
II. Problem solving (150 min)	Problem solving rationale and model, focused on factors that can be influenced at the workplace Validating context for effective problem solving Case-based skills training	II. Problem solving (120 min)	Problem solving rationale and model, focused on factors that can be influenced at the workplace Assertive communication for effective problem solving Group-discussions based on experiences
2 weeks to work on home assignments	Applied validation and problem solving with employees		Assertive communication and validation. Applied problem solving at work
III: Skills training and maintenance (150 min)	Role-play and skills training based on individual experiences Individual plan for maintenance	III. Skills training and maintenance (120 min)	Group discussions and re-evaluation based on individual experiences

The aim of the ECO program is to target key psychosocial factors at the workplace, specifically supervisor-employee interaction and their joint communication and problem-solving when managing pain and stress problems at work. The program is based on models of problem solving and supportive communication [21, 22]. The program includes skills training, by using cases, role-play, homework and reflection.

### Measures 

Sick leave data were collected from the Swedish Social Insurance Agency. Self-rating questionnaires (Swedish versions) were completed online via Örebro University’s secure survey system. The baseline assessments were sent out four weeks before intervention, the post intervention assessment was provided immediately after, and the follow-up 6-months after the intervention. Non-responders were reminded via e-mail and a maximum of two phone calls from a research coordinator. At the post intervention and 6-months follow-up, all participants received a movie ticket for completed questionnaires. Demographic information about age, sex, education level, social and occupational status were assessed at baseline. Program participation was assessed by self-report at post intervention. For supervisors, individuals with missing information on program participation were contacted through email and/or telephone calls.

The following outcomes were evaluated for the individual employees.

#### Psychoeducative Lectures (PE)

The active control intervention consisted of evidence-based psychoeducation about risk factors and self-management for pain and/or stress-related ill health [12, 13]. Participants were invited to 2 lectures of 1 h each, and received complementary information folders about pain and stress. The lectures were led by licensed psychologists at the occupational health care service facilities. The content of the intervention covers the biopsychosocial model of pain and stress-related ill-health, common misconceptions, risk factors for sick leave, and recommended self-management and information about workplace interventions. Both lectures were recorded and available “on-demand” via a secure web-portal together with downloadable information folders.

Both groups had access to usual care from the occupational health care service during the study.

#### Primary Outcome

The primary outcome was total number of days on sick leave during the 6-month time period after the intervention. Net days (all causes) of sick leave was used for register based sick leave data. Register based sick leave data were collected from the Swedish Social Insurance Agency, which manages sickness cash benefit for sick leave spells exceeding 14 days. Baseline data were collected for 6-months before intervention. For self-reported sick leave data, an item from the Work Ability Index was used at baseline and 6-months follow-up, where participants report days on sick leave during the last year: 0 days, 1–7 days, 8–24 days, 25–99 days, 100–356 days [24]. In addition, from the register data, the number of individuals with prevalence of a sick leave spell for the time period 6-months after the intervention versus before was used as a measure of sick leave outcomes [23].

#### Secondary Outcomes

The Work Ability Index (WAI) was used for self-rated work ability [24]. The WAI includes self-rated work ability in relation to demands of the work, and the individual’s current health status. The WAI score can be categorized as poor (7–27 points), moderate (28–36 points), good (37–43 points) and excellent (44–49 points) work ability. The WAI has been extensively used in working populations, and has acceptable reliability [25] and predictive validity for long-term sick leave [26].

Disability at work was measured by the Work Limitations Questionnaire -16 (WLQ), which has shown promising psychometric properties [27]. In the WLQ, the impact of health problems on occupational performance is measured, in this study pain and/or stress-problems specifically. An index scale 0–100 is calculated, where higher scores indicate more problems.

Pain-disability risk was measured using the Orebro Musculoskeletal Pain Screening Questionnaire (OMPSQ) [28, 29]. The OMPSQ covers sick leave, function in daily activities, psychological status, pain-related beliefs, and recovery expectations. A total score ranges from 2 to 210 points, with higher values corresponding to higher risk. A cut-off score of 90 indicates individuals at risk for long-term pain-related disability [29]. The OMPSQ has shown satisfactory reliability and predictive validity [28, 29]. In this study, additional options for pain locations were added (stomach, head, other), with the same scoring of the pain-location item, hence not changing the total score.

The Karolinska Exhaustion Disorder Scale (KEDS) [30] was used to measure symptoms of exhaustion disorder, as a proxy for risk for sick-leave due to stress problems. It covers consequences and symptoms of long-term stress during the past 2 weeks. The total score ranges from 0 to 54 with higher values reflecting more severe symptoms. A cut-off score of 19 has been shown to indicate exhaustion disorder [30], corresponding to the similar construct burn-out [31]. Reliability and validity of KEDS has been shown satisfactory [30].

The Perceived Stress Scale-10 (PSS-10) [32] was used to measure general symptoms of stress. In the PSS, respondents rate their perception of life events during the last month and total score ranges from 0 to 40 with higher values representing a high stress level. The short version (10 item) has shown good reliability and validity [32, 33].

Perceived health was measured using a visual analogue scale (VAS) where participants rated perceived health during the last 30 days. The scale was in digital format, horizontal and anchored with 0 = worst imaginable and 100 = best imaginable. Visual analogue scales have been evaluated extensively [34] and proven reliable in digital format [35].

Quality of life (QoL) was measured using the Brunnsviken Brief Quality of Life Questionnaire (BBQ) [36]. The BBQ is based on the overall life satisfaction conceptualization of QoL and covers satisfaction and importance of different life domains. The total score ranges from 0 to 96, where higher score indicates better outcome. The BBQ has shown high reliability and validity [36].

Perceived communication in terms of validation (to express understanding and acknowledge the validity in a person’s experience) and invalidation (the opposite) from the supervisor were measured using a modified 14-item version of Validating and Invalidating Response Scale (VIRS) [37], here adjusted to the supervisor-employee relationship. The two subscales, validation 0–36 with higher score indicating more validation from supervisor, and invalidation 0–20, with higher score indicating more invalidation, were used in reporting.

Perceived social support from supervisor was measured using two items from the short-version General Nordic Questionnaire for Psychological Factors at Work [38]: “If needed, can you get support and help with your work from your immediate superior?” and “Are your work achievements appreciated by your immediate superior?”. Items are calculated to a mean value 1–5 where higher scores indicate high perceived social support. The QPS is a reliable and valid measure [39].

### Statistical Analyses

On the continuous primary outcome sick leave (total number of days from register data) and secondary outcome variables (questionnaires), factorial repeated measure of variances with covariates (ANCOVA) were used, with the assumptions for analyses checked. The main outcomes were time (baseline/post intervention/6-months follow-up) * condition (ECO group/PE group), on each variable. Further, on the sick leave outcome, self-reported sick leave at 6-months follow-up was analyzed using chi-square test, to evaluate difference between conditions. The effect on number of individuals with prevalence of a sick leave spell, based on register data, was analyzed using hierarchical logistic regression, to evaluate impact of condition on sick leave, while controlling for baseline values and co-variates.

Results were analyzed on the individual level. In all analyses of outcomes, type of workplace was included as a co-variate, based on potential workplace level effects and the identification of interactions with outcome variables at baseline. Data assessment before or during the Covid-19 pandemic was also included as a co-variate. Intra-cluster correlation coefficients (ICC) were calculated for the outcomes, for the clusters of workplace-units. Based on low to moderate ICC values for the primary outcome, and a relatively small number of clusters, the cluster level of randomization units was not further accounted for in the analyses.

Analyses were based on an intention to treat (ITT) approach, including all participants with baseline assessments available. An ITT approach, with imputation of missing data, reduces the risk of biased and incorrect inference in randomized controlled trials [40]. The multiple imputation method was used to handle missing data, a commonly used and valid method [41]. The level of missing data and pattern of missingness in data indicated that 21.7% of values on outcome variables were missing. Further, Little’s missing completely at random (MCAR) test indicated that the data was missing completely at random (X^2^(1565) = 1534.771, p = 0.70). Multiple imputation with 5 iterations was used, a method which is suggested to result in unbiased estimates when the proportion of data missing is limited (less than 40%) in RCT’s [40]. Pooled mean values on the outcome variables are reported. Analyses of outcomes were performed on the complete data as well as on the 5 imputed data-sets, and among these, the range of results from lowest to highest p-value are reported.

In addition to the ITT approach, analyses were performed on a sample of per-protocol participants (participating in the interventions). Results did not differ, and analyses on the ITT sample are reported together with results from individuals with complete data. Due to differences between conditions on stress-related ill-health at baseline, additional analyses were performed to rule out this potential impact. A matching was performed based on propensity scores. Results from the analyses on the matched sample did not differ, and results from the original sample are reported.

All statistics were conducted using SPSS version 27.0, IBM statistical software. For analyses of group differences at baseline, and analyses of non-responders, t-tests or chi-square test were used. Throughout, comparisons were two-tailed and were treated as statistically significant at the level of p < 0.05.

## Results

### Participant Flow

**Fig. 1:**
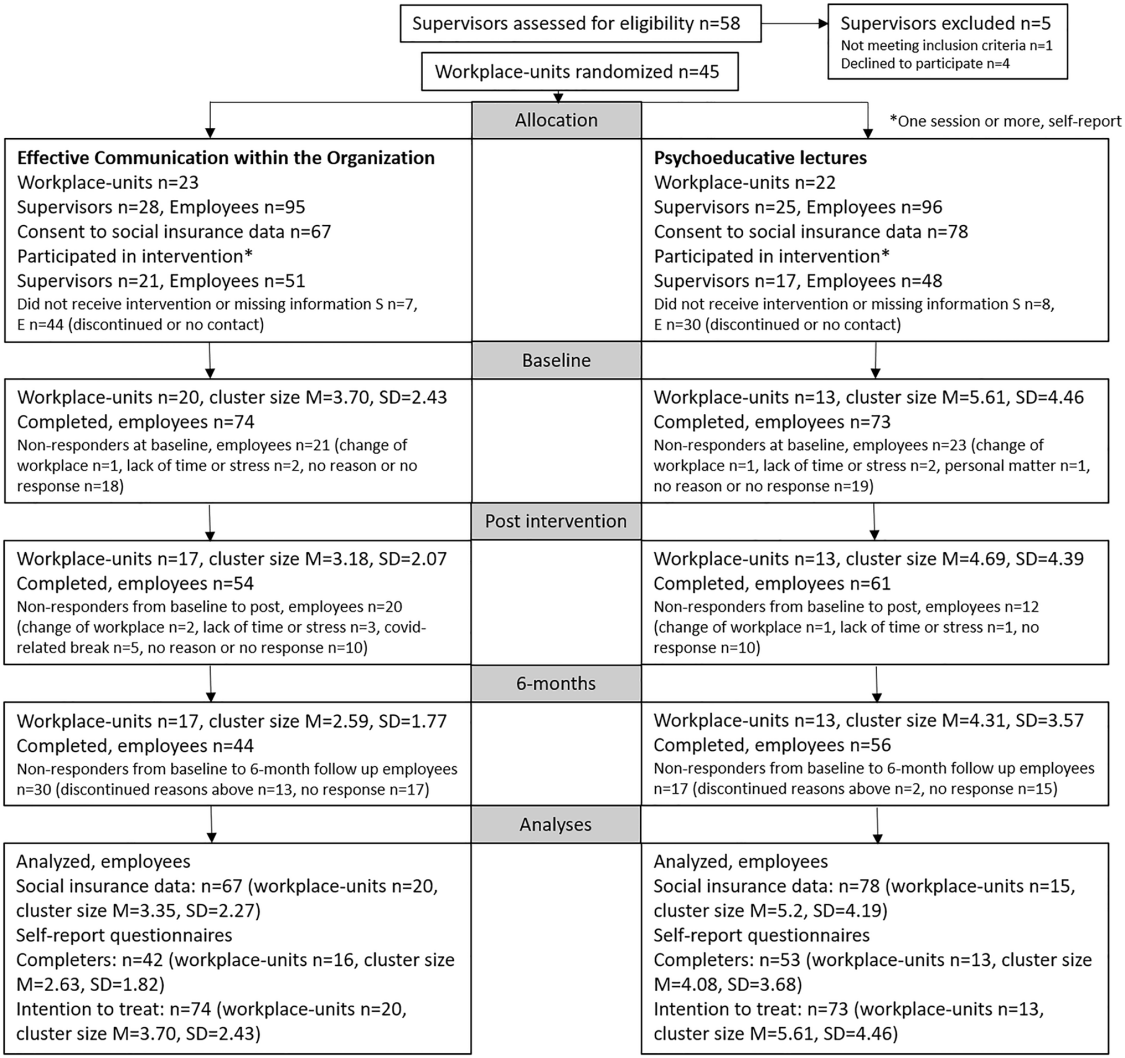
Flowchart of recruitment, available data and study participation

Figure 1 shows a flowchart of the participants. A total of 58 supervisors were assessed for eligibility, and 53 supervisors were included and randomized, distributed on 45 workplace-units. In the experimental condition (ECO), 95 employees were recruited and in the control condition (PE) 96 were recruited, i.e. a total of 191 employees. Among these, 145 gave consent to extraction of social insurance data. A majority of the supervisors participated actively in the intervention programs (75% in the ECO and 68% in the PE group), resulting in a large proportion (79%) of the employees having a supervisor with active participation. Among the employees, about 50% participated actively in the interventions (54% in the ECO and 50% in the PE groups). For employees the amount of missing data on program participation was high. There were no differences between conditions in participation rates among supervisors or employees.

### Non-responders

A total of 147 employees filled out the baseline questionnaires. There were no differences in attrition rate from recruitment to baseline between the conditions, but attrition before baseline was significantly higher among health care services, X^2^ (2, N = 191) = 13.26, p = 0.001. Also, there were no differences between conditions regarding consent to social insurance data.

A total of 115 of employees filled out the post intervention questionnaires and a total of 100 filled out the 6-months follow up. There was more attrition from baseline to 6-months follow up in the ECO group X^2^ (1, N = 147) = 5.03, p = 0.025. Among those with baseline assessment data available, non-responders at 6-months follow up did not differ from responders regarding demographic or outcome variables. A similar pattern was observed among the 27 participants with missing on both post and 6-months, with a tendency to larger attrition in the ECO group X^2^ (1, N = 147) = 3.53, p = 0.06, and also a higher proportion missing for those who participated in the intervention March-September (paused) 2020, X^2^ (3, N = 147) = 10.90, p = 0.01.

### Participants characteristics

Characteristics of employees with baseline assessment is reported in Table 2. The majority of employees were women with Swedish nationality, and health care services were the most common types of workplaces. Pain in at least one location was reported by 89% of the employees. In total, 30% of the employees were above the cut-off for risk of long-term pain-related disability at baseline, and 61% were above the cut-off of stress symptoms indicating exhaustion disorder. Employees in the ECO group reported higher levels of stress symptoms compared to the PE group, as can be seen in Table 2. There were no differences between conditions on outcome variables at baseline. In total, 40% of the participants had their intervention period during the pandemic, and 75% had data assessment during the pandemic. Intra-cluster correlation coefficients (ICC) was analyzed for the cluster level randomized workplace-units. For the primary outcome total days on sick leave, ICC was 0.114 at baseline and 0.002 at 6-months follow-up. For the secondary outcomes, ICC ranged between 0.119 and 0.319 across measures and assessment points.

**Table 2:** Characteristics of employees at the baseline assessment

Measure	ECO	PE	Between-group comparisons
N	74	73	
Gender, n women (%)	68 (91.9)	71 (97.2)	X^2^ (1, N = 147) = 2.06 p = 0.151
Age, mean (SD)	43.3 (10.2)	43.4 (10.3)	t(145) = 0.06, p = 0.953
Nationality, n Swedish (%)	65 (87.8)	69 (94.5) (n = 72)	X^2^ (3, N = 146) = 3.09 p = 0.378
Years at current work, mean (SD)	8.0 (7.5)	8.3 (8.9)	t(145) = 0.13, p = 0.895
Percent work time, mean (SD)	92.6 (14.1) (n = 73)	90.9 (12.6)	t(144) = − 0.80 p = 0.428
Highest education, n (%)			X^2^(2, N = 147) = 6.12 p = 0.047*
Middle or high school	13 (17.6)	16 (21.9)	
Vocational education	16 (21.6)	27 (40.0)	
University, bachelor or above	45 (60.8)	30 (41.0)	
Type of workplace, n (%)			X^2^(2, N = 147) = 9.08 p = 0.011*
Health care services	37 (50.0)	39 (53.4)	
Administrative departments	24 (32.4)	10 (13.7)	
Schools and services	13 (17.6)	24 (32.9)	
Pain prevalence^a^, n yes (%)	64 (86.5)	67 (91.8)	X^2^ (1, N = 147) = 1.06 p = 0.303
Pain intensity^b^, mean (SD)	4.33 (2.51) (n = 64)	4.52 (2.16) (n = 67)	t(129) = 0.48 p = 0.635
Pain related ill-health: risk of long-term pain disability^c^, n (%)	25 (37.3) (n = 67)	19 (28.4) (n = 67)	X^2^ (1, N = 147) = 1.22 p = 0.270
Stress related ill-health: indication of exhaustion disorder^d^, n (%)	51 (72.9) (n = 70)	39 (54.2) (n = 72)	X^2^(2, N = 142) = 5.34 p = 0.021*

*N* number of responses, *SD* standard deviation

^a^Any pain location, as reported in the Orebro Musculoskeletal Pain Screening Questionnaire (OMPSQ)

^b^Mean rating of pain during the last week, 0–10

^c^Above cut-off 90 OMPSQ

^d^Above cut-off 19 Karolinska Exhaustion Disorder Scale

*Significance at p < 0.05

### Effects on Outcome Variables

#### Primary Outcome Sick Leave

On total days on sick leave from register data, ANCOVA analysis displayed no effect of condition over time, F (1,145) = 2.11, p = 0.149, for sick leave during 6-months after the intervention compared to before. Self-report of sick leave during the last year did not differ between conditions at 6-months follow-up, X^2^ (4, N = 98) = 3.74 p = 0.442, or at baseline. For numbers of individuals with a sick leave spell, there was no effect of conditions on prevalence during 6-months follow-up after the intervention, OR 0.83 (p = 0.70, CI 0.31–2.18), when controlling for type of workplace, data assessment during the Covid-19 pandemic and prevalence of sick leave spell before the intervention. Descriptive values for sick leave outcomes can be found in Table 3.

**Table 3:** Sick leave outcomes for individuals in the Effective Communication within the Organization (ECO) and the psychoeducative lectures (PE) group for register data and self-report

Measure	ECO		PE	
Sick leave from register data during a 6-months period^a^	Before intervention (n = 67)	After intervention (n = 67)	Before intervention (n = 78)	After intervention (n = 78)
Total days on sick leave, M (SD)	8.65 (20.34)	8.93 (27.20)	3.87 (13.73)	9.42 (34.86)
Prevalence of a sick leave spell, n (%)	14 (20.9)	14 (20.9)	8 (10.3)	11 (14.1)
Self-reported sick leave during the last year ^b^, %	Baseline (n = 74)	6-months follow-up (n = 43)	Baseline (n = 73)	6-months follow-up (n = 55)
0 days	12.2	16.3	25.0	18.2
1–7 days	33.8	27.9	38.2	43.6
8–24 days	28.4	39.5	19.7	23.6
25–99 days	23.0	11.6	11.8	10.9
100–365 days	2.7	4.7	1.3	3.6

^a^Data from the Swedish Social Insurance Agency, during a 6-month time period before or after intervention

^b^Item from the Work Ability Index

#### Secondary Outcomes

The repeated measures ANCOVAs displayed no significant interaction effects between condition and time on work ability, work limitations, pain disability risk, exhaustion symptoms, perceived stress, perceived health, quality of life, and perceived communication from supervisor (validation or invalidation), meaning no group differences over time were observed. On perceived social support from supervisor, there was a significant difference between ECO and PE when analyzing complete data, however no significant interactions in the intention to treat group with the imputed data sets. Mean values and standard deviations for completers (N = 93) and pooled mean values for the intention to treat group (N = 147) can be found in Table 4. Statistics for the ANCOVA analyses can be found in Table 5.

**Table 4:** Means and standard deviations on outcome variables for employees at baseline, post intervention and 6-months follow up per group, for completers and intention to treat (pooled)

Measure	ECO completers	PE completers	ECO ITT (pooled)	PE ITT (pooled)
	Mean (SD)	Mean (SD)	Mean	Mean
Work ability^a^	n = 27	n = 40	n = 74	n = 73
Baseline	36.11 (6.74)	36.49 (7.03)	35.88 (6.21)	37.54 (6.52)
Post intervention	35.91 (7.13)	36.63 (7.08)	35.54 (7.17)	37.64 6.66)
6-months	37.57 (6.99)	37.00 (8.46)	36.09 (8.01)	37.94 (7.75)
Work limitations^b^	n = 37	n = 49	n = 74	n = 73
Baseline	29.48 (13.73)	26.95 (14.68)	30.50 (14.63)	27.06 (14.21)
Post intervention	30.21 (14.31)	24.77 (13.68)	30.72 (18.47)	26.33 (15.17)
6-months	27.83 (14.31)	23.96 (16.52)	28.09 (17.46)	23.67 16.64)
Pain-disability risk^c^	n = 31	n = 40	n = 74	n = 73
Baseline	73.24 (36.34)	67.96 (33.31)	75.60 (42.40)	71.17 (34.66)
Post intervention	71.00 (46.00)	60.18 (36.80)	70.71 (48.52)	64.00 (39.31)
6-months	63.80 (51.64)	53.38 (43.39)	68.23 (56.42)	58.39 (44.37)
Exhaustion symptoms^d^	n = 39	n = 49	n = 74	n = 73
Baseline	23.82 (9.46)	19.76 (9.39)	22.30 (9.27)	19.47 (9.05)
Post intervention	22.44 (10.66)	19.31 (10.36)	21.36 (10.34)	19.17 (10.50)
6-months	20.97 (11.12)	17.51 (10.38)	19.27 (11.26)	17.06 (10.75)
Perceived stress^e^	n = 38	n = 45	n = 74	n = 73
Baseline	20.66 (6.24)	17.67 (6.60)	19.31 (6.13)	18.12 (6.31)
Post intervention	19.61 (5.80)	17.44 (5.21)	18.75 (5.93)	17.69 (5.63)
6-months	17.89 (6.96)	16.64 (5.90)	17.19 (7.27)	16.55 (6.21)
Perceived health^f^	n = 41	n = 53	n = 74	n = 73
Baseline	54.10 (23.64)	61.25 (23.17)	53.65 (23.45)	60.15 (22.70)
Post intervention	55.22 (24.57)	59.19 (23.65)	54.32 (25.48)	58.92 (24.41)
6-months	60.20 (20.29)	64.53 (25.28)	60.64 (23.33)	64.82 (25.85)
Quality of life^g^	n = 37	n = 46	n = 74	n = 73
Baseline	55.81 (21.67)	65.78 (21.28)	58.67 (20.60)	63.87 (21.53)
Post intervention	57.92 (23.90)	64.09 (18.84)	59.47 (22.59)	63.41 (20.73)
6-months	57.89 (22.88)	65.89 (23.77)	60.89 (24.11)	65.94 (25.29)
Perceived communication and support from supervisor:				
Validation^h^	n = 40	n = 51	n = 74	n = 73
Baseline	27.35 (8.49)	26.35 (7.87)	26.93 (8.28)	25.89 (7.75)
Post intervention	26.25 (9.70)	25.55 (7.67)	25.52 (9.42)	24.75 (7.73)
6-months	26.85 (7.76)	24.53 (6.68)	26.06 (8.12)	23.98 (7.39)
Invalidation^i^	n = 36	n = 48	n = 74	n = 73
Baseline	3.06 (3.41)	2.81 (3.60)	3.19 (3.64)	2.96 (3.47)
Post intervention	3.61 (3.61)	3.04 (3.41)	3.54 (4.08)	3.12 (3.46)
6-months	3.64 (3.74)	3.21 (3.25)	3.59 (4.06)	3.58 (4.00)
Social support^j^	n = 41	n = 52	n = 74	n = 73
Baseline	3.34 (1.13)	3.53 (1.05)	3.45 (1.06)	3.44 (1.08)
Post intervention	3.51 (1.08)	3.50 (0.95)	3.52 (1.07)	3.40 (1.00)
6-months	3.68 (1.07)	3.43 (0.86)	3.69 (1.13)	3.46 (0.98)

^a^Work Ability Index 7–42 higher scores better work ability

^b^Work Limitation Questionnaire index 0–100 higher scores more problems

^c^Orebro Musculoskeletal Pain Screening Questionnaire 2–210 higher score more risk

^d^Karolinska Exhaustion Disorder Scale 0–54 higher score more symptoms

^e^Perceived Stress Scale-10 0–40 higher score more perceived stress

^f^Visual Analogue Scale of Health 0–100 higher score better health

^g^Brunnsviken Brief Quality of Life Scale 0–96 higher score better life satisfaction

^h^Validation subscale 0–36 higher score more validation from supervisor

^i^Invalidation subscale 0–20 higher score more invalidation from supervisor

^j^Social support from supervisor 1–5 higher scores more perceived support

**Table 5:** Between-group analyses on outcome variables, with repeated measures statistics, for completers N = 95 and intention to treat N = 147 (imputed data)

Measure	ANCOVA (time × condition)		
	Completers	Imputed data, range (p-value)	
		Lowest	Highest
Work ability	F(1.82,67) = 0.09, p = 0.901	F(1.80,147) = 1.16, p = 0.310	F(1.80,147) = 0.10, p = 0.886
Work limitations	F(2,86) = 0.41, p = 0.960	F(1.88,147) = 0.30, p = 0.729	F(1.76,147) = 0.014, p = 0.978
Pain-disability risk	F(2,71) = 0.22, p = 0.802	F(2,147) = 1.922, p = 0.148	F(1.78,147) = 0.25, p = 0.752
Exhaustion symptoms	F(2,88) = 0.72, p = 0.488	F(1.78,147) = 1.42, p = 0.245	F(1.79,147) = 0.11, p = 0.871
Perceived stress	F(2,83) = 1.21, p = 0.301	F(1.82,147) = 1.542, p = 0.217	F(1.75,147) = 0.14, p = 0.838
Perceived health	F(2,94) = 0.70, p = 0.499	F(2,147) = 0.68, p = 0.508	F(2,147) = 0.22, p = 0.801
Quality of life	F(2,83) = 0.88, p = 0.418	F(1.77,147) = 1.42, p = 0.245	F(1.85,147) = 0.11, p = 0.882
Perceived communication and support from supervisor			
Validation	F(1.73,91) = 0.29, p = 0.717	F(1.68,147) = 2.56, p = 0.089	F(1.76,147) = 0.126, p = 0.849
Invalidation	F(1.62,84) = 0.19, p = 0.778	F(1.67,147) = 1.68, p = 0.193	F(1.83,147) = 0.02, p = 0.970
Social support	F(2,93) = 3.58, p = 0.030*	F(1.91,147) = 2.12, p = 0.124	F(2,147) = 0.57, p = 0.564

Co-variates in the model were type of workplace and data assessment during the Covid-19 pandemic. Condition = intervention group ECO or PE

*Significance at p < 0.05

## Discussion

This study evaluated the effects of a preventive psychosocial intervention on employees reporting pain and stress-related ill-health compared to an active control intervention. No effects for the brief ECO intervention, relative to the control, were observed on the primary or secondary outcomes. It is possible the changed protocol due to the Covid-19 pandemic may have affected the results, but it is not possible to determine this. An indication of effect from the ECO on perceived social support from supervisor was noted, but this could have been due to chance and would need to be replicated. Preventive intervention trials are challenging in terms of estimations of expected effect size, power calculations, and identifying and including a target population at risk and this issue merits further discussion (see below).

The positive effect of an intervention similar to the ECO program, reported in the previous study [11], was not replicated here. One difference between projects was the inclusion of an active control group, which might have influenced the results. The other important difference between the two projects are the selection of samples. The earlier study used a structured and validated screening instrument, detecting risk for long-term pain-related disability, whereas this study targeted an extended population of individuals with self-reported pain and stress-related ill-health, increasing heterogeneity. The sample in the current study proved to have a much lower pain-disability risk level, compared to the earlier study. Only about one third of employees in the current study were at risk for long-term pain-related disability, even though a vast majority reported prevalence of pain.

The lower levels of pain-disability, as compared to the earlier study, might have affected how the intervention was received as well as effect sizes. Sample size did not allow for subgroup analyses of individuals at risk. Another indication that the sample was not a high risk population are results on moderate to good work ability according to the WAI, slightly lower than in a general population [26]. It is also known that early preventive interventions, such as cognitive-behavioral interventions or patient education, display limited value for low risk groups [12, 42, 43]. Our results appear consistent with these earlier findings. In addition, a longer follow-up period would have been preferable, specifically to be able to detect impact on sick leave.

Another possible explanation for our results is the brevity of the ECO intervention. Previous research indicates the workplace is an important arena for prevention of ill-health, and there are numerous examples of benefits on work disability of workplace involvement, and problem solving at the workplace for employees with work limitations [3–6, 44]. However, research on interventions focused on supervisor training only (in contrast to workplace interventions tailored to the employee at risk of more sick leave) suggests these have limited effectiveness in enhancing employee health or well-being [45, 46].

Employees in this study reported a relatively high prevalence of stress symptoms, which might have implications for the content of the interventions. Similar as for pain conditions, there is support for cognitive behavioral therapy as a treatment for stress-related diagnoses, and in addition as a form of occupational stress management [47, 48]. Workplace involvement for individuals on sick leave due to stress show variable outcomes on return to work [5, 44, 49, 50]. Even less is known about early interventions and managing psychosocial workplace factors for individuals at risk of stress-related ill-health. Results from this study indicate that even though pain and stress-problems often co-occur, sub-group analyses based on individual characteristics might be needed, requiring larger samples for statistical power. It should also be noted that almost all participants in this study were women, which largely corresponds to employees in these sectors, but it should be taken in consideration when contextualizing the results.

### Methodological Considerations

The power calculation, based on self-reported sick leave from the previous project, estimated that a sample of 182 employees was needed. A limitation is that the sample size calculation did not include estimation of design effect, which resulted in an under-powered study. The recruitment resulted in 191 employees, however 145 (76%) gave consent to social insurance data and 147 filled out the baseline assessment, which further reduced power for primary and secondary outcomes. Consequently, sample size was also low in analyses of per-protocol participants and matched participants, and sample size did not allow for sub-group analyses for individuals at risk, types of workplace nor for Covid-19 pandemic.

The number of non-responders among employees was high at all time-points of data assessment, with a retention rate of 52.4% from recruitment to 6-months follow-up. It should be noted these figures are much higher than those reported in the previous, similar study [11]. The large amount of missing data throughout the study may introduce a threat to internal validity and affect outcomes. The large number of questionnaires might have been burdensome for the respondents, and should preferable be reduced in future studies. Due to differences in results on completers and the ITT group, both are reported.

Conducting the study during the Covid-19 pandemic was challenging, and ideally, the program should be re-run during ordinary conditions. The pandemic affected recruitment, participation and delivery of intervention, which might have affected integrity and effectiveness. The pandemic might also potentially had different impact for different individuals and workplaces. Interactions on outcome variables was found for participation during the pandemic, but also for type of workplace, and they were both included as co-variates in analyses.

Another methodological limitation was differences between employees in the ECO and the PE group, which was handled by complementary analyses on matched participants. The unbalanced groups seem to be associated with the different distribution of types of workplace between conditions, and hence might be related to the cluster aspects of the randomization and recruitment process. In future projects, the cluster design could be better handled with a larger power or a stratification of types of workplaces. An alternative analytic approach would be to use multilevel analyses, adding the cluster level in the analyses. In summary, for future similar projects, we recommend larger sample sizes, longer time for follow-up, effort to include a more diverse sample regarding gender, and actions to increase study participation and response rate.

## Conclusions

This study evaluated the effects of brief preventive interventions for employees with pain and stress-related ill-health. The study was intended to replicate and extend the findings from an earlier similar project for individuals with musculoskeletal low back pain, but this time with a more heterogeneous sample not selected on the basis of assessed psychosocial risks. We compared a skills-based psychosocial intervention, Effective Communication within the Organization (ECO), to psychoeducative (PE) lectures. The results displayed no effects of the ECO on employees regarding register based or self-reported sick leave or secondary outcomes. The positive outcomes from the previous study were not replicated, and preventive effects of the ECO program in this population were not supported. It should be kept in mind that the pandemic did affect the study procedures and this may have affected our results, but there is no way to determine this. Another important aspect is that employees in this study was not a high-risk population in psychosocial risk levels. When considered in combination with a similar previous study, these findings suggest the importance of selecting participants for these interventions based on their assessed psychosocial risk profiles. Further research on communication and support at the workplace and its interactions with employee health and work environment is warranted.

## Data Availability

The data presented in this study are available on request.
